# Prognostic value of postoperative anti-thyroglobulin antibody in patients with differentiated thyroid cancer

**DOI:** 10.3389/fendo.2024.1354426

**Published:** 2024-04-24

**Authors:** Yihan Zhao, Zhuanzhuan Mu, Dongquan Liang, Teng Zhang, Xin Zhang, Di Sun, Yuqing Sun, Jun Liang, Yansong Lin

**Affiliations:** ^1^ Department of Nuclear Medicine, Beijing Key Laboratory of Molecular Targeted Diagnosis and Therapy in Nuclear Medicine, State Key Laboratory of Complex Severe and Rare Diseases, Peking Union Medical College (PUMC) Hospital, Chinese Academy of Medical Sciences & PUMC, Beijing, China; ^2^ Department of Nuclear Medicine, The First Affiliated Hospital of Anhui Medical University, Hefei, China; ^3^ Department of Psychology, Pepperdine University Graduate School of Education and Psychology, Los Angeles, CA, United States; ^4^ Department of Nuclear Medicine, Beijing Chaoyang Hospital, Capital Medical University, Beijing, China; ^5^ Department of Oncology, Peking University International Hospital, Beijing, China

**Keywords:** differentiated thyroid cancer, anti-thyroglobulin antibody, biomarker, prognosis, persistent/recurrent disease

## Abstract

**Purpose:**

Postoperative thyroglobulin (Tg) generally serves as a biomarker to monitor the recurrence or persistence of differentiated thyroid cancer (DTC), whereas it constrains to interference from anti-thyroglobulin antibody (TgAb). This study aimed to determine the value of postoperative TgAb as a surrogate for monitoring tumor status in DTCs with positive TgAb after successful radioactive iodine (RAI) remnant ablation.

**Methods:**

We retrospectively enrolled DTC patients with positive (≥40 IU/mL, Roche) postoperative TgAb measurements. An index of TgAb change (ΔTgAb) was defined to describe the TgAb decrease rate. DTC status was defined as either no evidence of disease (NED) or persistent/recurrent disease (PRD). Univariate and multivariate binary logistic analyses were used to identify the independent risk factors of PRD. Receiver operating characteristic (ROC) curves were performed to determine the optimal cutoff values of each risk factor, and DeLong’s test was conducted to compare their predictive powers. Kaplan–Meier curves were used to assess the impact of different TgAb trends in the first year on progression-free survival.

**Results:**

Of the 232 patients enrolled, the median diagnosis age was 34 years (range, 18–62 years), with a male-to-female ratio of 1:4.66 (41/191). Among them, after a median follow-up of 44 months (range, 4–128 months),183 (78.87%) patients were evaluated as NED, while the other 49 (21.12%) had either persistent (*n* = 25) or recurrent disease (*n* = 24). Multivariate regression showed that ΔTgAb (*P* < 0.001) and lymph node metastasis (LNM) rate (*P* = 0.009) were independently relevant to the presence of PRD, with optimal cutoff values of 47.0% and 35.1%, respectively. It is important to note that there is a high negative predictive value (96.93%) of ΔTgAb with the cutoff of 47.0%. DeLong’s test showed that ΔTgAb alone and the combination of ΔTgAb and LNM rate were significantly greater than the isolated LNM rate (both *P* < 0.001) in predicting NED, while there was no statistical difference of the predictive power between ΔTgAb and the combination (*P* = 0.203). Additionally, patients with ΔTgAb >47.0% had longer progression-free survival than those with ΔTgAb ≤47.0% (not reached vs. 50 months, *P* < 0.001), and those with ΔTgAb >47.0% or negative conversion within the first year after RAI ablation had longer progression-free survival.

**Conclusion:**

Our study suggested that ΔTgAb could serve as a valuable indicator of disease status in DTC patients with positive TgAb. A ΔTgAb of >47.0% is conducive to identify those with NED and may help to obviate their overtreatment. The decrease rate and negative conversion of TgAb in the first year were good predictors of disease-free survival in patients.

## Introduction

Differentiated thyroid cancer (DTC) is the most prevalent endocrine malignancy, the majority of which are papillary thyroid cancer (PTC) and follicular thyroid cancer (FTC) with an excellent prognosis (1). Currently, total or near-total thyroidectomy, postoperative selective radioactive iodine (RAI/^131^I), and long-term thyroid-stimulating hormone (TSH) suppression are commonly used as the first-line clinical treatments for patients with DTC (2). Biochemical measurements in combination with imaging examinations such as cervical ultrasound (US), chest computed tomography (CT), and radioiodine diagnostic whole-body scan (DxWBS) are considered the standard of care for the management of DTC. Thyroglobulin (Tg) is usually used as a sensitive and convenient biochemical tumor marker after initial therapy. Nevertheless, its accuracy may be interfered by anti-thyroglobulin antibody (TgAb) in up to 25% of DTC patients (3). Moreover, a dilemma emerged in such patients as to how to biochemically monitor the disease status during follow-up. In the 2015 American Thyroid Association (ATA) guidelines, measuring both serum TgAb and Tg levels simultaneously is recommended for DTCs during follow-up (4). Theoretically, after the removal of all thyroid tissue by total thyroidectomy and ^131^I therapy in patients with DTC, Tg should be cleared, leading to a gradual decrease of TgAb until it disappears (5). Therefore, a falling TgAb trend over time often indicates the remission or improvement of the disease (6). However, as TgAb is a marker reflecting immuno-reaction, depending on the different immuno-backgrounds of patients, it remains quite illusive for us to define and expect the definite change as Tg. Several studies have demonstrated that in DTC patients with TgAb reduced slowly, long-term stable or increasing TgAb levels appear to have a varying degree of increased risk of persistent or recurrent disease (PRD) compared to those exhibiting a considerable reduction (7–13). However, so far, the dynamic changes of TgAb at postoperative follow-up and TgAb quantitative analysis in relation to the clinical outcomes of TgAb-positive DTC patients after RAI therapy remain not yet well addressed. Our objective was to evaluate the clinical value of change in TgAb as a predictor of disease status during the follow-up of patients after thyroid ablation.

## Patients and methods

### Study population

**Figure 1:**
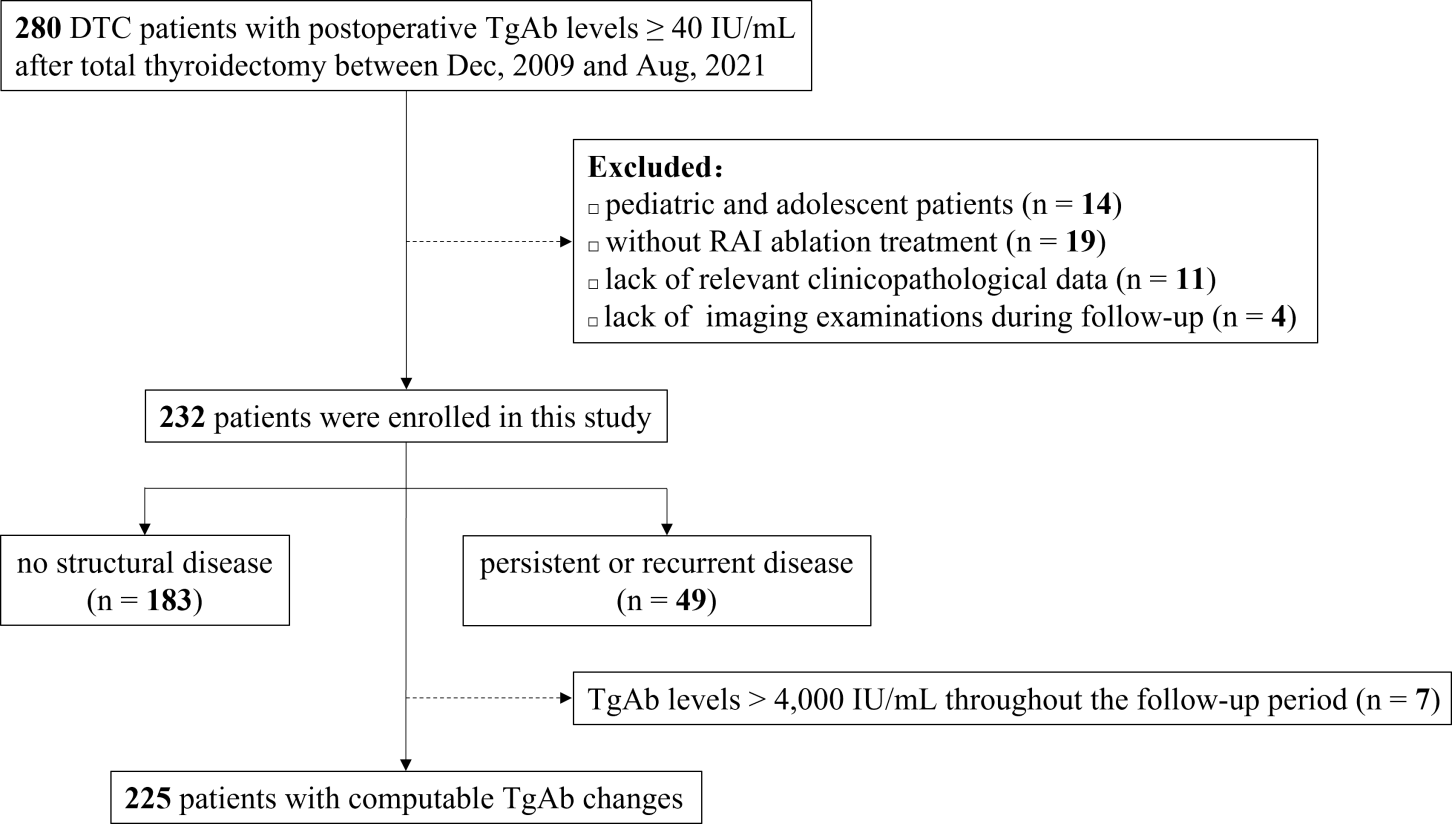
Case screening process of the study. DTC, differentiated thyroid cancer; TgAb, anti-thyroglobulin antibody; RAI, radioactive iodine.

In this retrospective study, we screened the records of DTC patients at Peking Union Medical College Hospital between December 2009 and August 2021. The inclusion criteria were as follows: (1) the patients underwent total or near-total thyroidectomy and were pathologically diagnosed as DTC, including PTC and FTC, (2) the patients received RAI ablation, (3) detected with positive postoperative TgAb measurements, and (4) at least two TgAb measurements available during follow-up. On the other hand, individuals were excluded if they qualify under any of the following: (1) pediatric and adolescent patients aged <18 years at the time of diagnosis, (2) lack of relevant clinicopathological data such as histology and primary tumor size, and (3) lack of imaging examinations such as cervical ultrasound, computed tomography, or ^131^I-whole body scan (WBS) during follow-up. The screening process is shown in **Figure 1**. In total, 232 patients were enrolled in the study. In seven cases, the TgAb levels were measured consistently above the upper limit of detection (>4,000 IU/mL) during follow-up, which could not determine TgAb change trends. Thus, 225 patients were finally included when computing ΔTgAb, which was calculated by the following equation: (TgAb level at initial follow-up - TgAb level at final follow-up)/(TgAb level at initial follow-up) × 100%. A positive value indicates a decrease of TgAb level at final follow-up relative to the initial, and a negative value indicates an increase.

### Follow-up strategy and clinical outcome

In accordance with the ATA guidelines, all patients were given inhibitory doses of levothyroxine after surgery and iodine treatment, and then they received regular follow-up for a long term (4). The follow-up examinations included serum, ultrasound, and radiological examinations. Tg and TgAb concentrations were measured at each visit, while imaging procedures such as neck US and chest CT were performed on schedule. If a suspicious lesion is detected during follow-up, further imaging such as DxWBS and/or positron emission tomography/computed tomography (PET/CT) may be performed, and pathology would be obtained to confirm the lesion when the patient received further surgery or biopsy.

The clinical outcome of each patient was determined by a pathology report and/or imaging findings during follow-up. The patients were divided into no evidence of disease (NED, no suspicious structural/functional findings) or persistent or recurrent disease (PRD). In addition, progressive disease, including all recurrent diseases and part of a persistent disease, was evaluated in accordance with the Response Evaluation Criteria in Solid Tumors (RECIST), version 1.1. Accordingly, progression-free survival (PFS) was defined as the time interval from successful RAI remnant ablation to either disease progression or the latest visit for those with NED or non-progressive persistent disease.

The follow-up time of DTC in clinical practice is generally long and might vary tremendously between patients. Meanwhile, in order to provide a more convincing reference method for TgAb in clinical follow-up, according to Kim et al. (7), we chose the first year after RAI ablation as the time interval to stratify the decrease rate and status of TgAb to assess the impact of different TgAb decrease rates and status of negative conversion in the first year on PFS.

### Laboratory studies

Serum Tg and TgAb levels were determined by electrochemiluminescence immunoassay (provided by Roche Diagnostics GmbH, Mannheim, Germany) with measurement ranges of 0. 04 to 500 ng/mL and 10 to 4,000 IU/ml, respectively. Serum TgAb titers ≥40 IU/mL were defined as positive TgAb, which might interfere with Tg measurement (14, 15). TSH was determined by chemiluminescence immunoassay (provided by Siemens Healthcare Diagnostics Inc., New York, NY, USA) with a detection range of 0.04 to 150 μIU/mL. Values above the ranges were reported as >500 ng/mL, >4,000 IU/mL, and >150 μIU/mL for Tg, TgAb, and TSH, respectively.

### Statistical analysis

Continuous variables of the two groups conforming to normal distribution were represented as mean ± standard deviation (SD) and compared by Student’s *t*-test, while non-normal variables were reported as median (range) and compared by Mann–Whitney *U*-test. Categorical variables were expressed as absolute numbers and percentage frequency distribution, compared by chi-square test or Fisher’s exact test. Binary logistic regression analyses were conducted to identify the risk factors of PRD, and variables with *P <*0.05 in the univariate logistic analysis were entered into a multivariate logistic analysis. For statistically significant indicators, receiver operating characteristic (ROC) analyses were conducted to evaluate the predictive values and identify the optimal cutoff values. The indicators’ areas under the ROC curve (AUCs) were also compared by DeLong’s test to estimate the predictive power. Kaplan–Meier curves and log-rank tests were used to assess the impact of different TgAb trends in the first year on PFS. All hypothesis tests were two-sided with *P <*0.05 indicating statistically significant differences.

## Results

### Clinical characteristics

Of the 232 patients eventually included in this study, 227 were diagnosed as PTC and five as FTC, with a median diagnosis age of 34 years (range, 18–62 years) and a male-to-female ratio of 1:4.66 [41 (17.67%)/191 (82.33%)]. At a median follow-up of 44 months (range, 4–128 months), 183 (78.88%) patients were identified with NED, while the other 49 (21.12%) had either persistent (*n* = 25/49, 51.02%) or recurrent disease (*n* = 24/49, 48.98%) with disease progression (defined by RECIST criteria) noticed in 30. The clinical characteristics of the study population are listed in **Table 1**. Maximal tumor size, capsular invasion, lymph node metastasis (LNM) rate, N stage, and cumulative RAI dose were statistically different between the NED and PRD groups. The LNM rate refers to the percentage of pathologically confirmed metastatic LNs accounted for surgically removed LNs.

**Table 1:** Clinical characteristics of 232 differentiated thyroid cancer patients with positive TgAb.

Characteristics	Total (*N* = 232)	NED (*N* = 183)	PRD (*N* = 49)	*P* value
Age at diagnosis (years)				0.911
Median	34	34	33	
Range	18–62	18–61	18–62	
Gender				0.886
Male	41 (17.67%)	32 (17.49%)	9 (18.37%)	
Female	191 (82.33%)	151 (82.51%)	40 (81.63%)	
Histology				0.951
Papillary	227 (97.84%)	179 (97.81%)	48 (97.96%)	
Follicular	5 (2.16%)	4 (2.19%)	1 (2.04%)	
Maximal tumor size (cm)				0.040^*^
Median	1.15	1	1.4	
Range	0.2–5.0	0.2–5.0	0.2–5.0	
Bilaterality				0.763
Yes	81 (34.91%)	63 (34.43%)	18 (36.73%)	
No	151 (65.09%)	120 (65.57%)	31 (63.27%)	
Multifocality				0.233
Yes	115 (49.57%)	87 (47.54%)	28 (57.14%)	
No	117 (50.43%)	96 (52.46%)	21 (42.86%)	
Hashimoto’s thyroiditis				0.900
Yes	126 (54.31%)	99 (54.10%)	27 (55.10%)	
No	106 (45.69%)	84 (45.90%)	22 (44.90%)	
Capsular invasion				0.004^*^
Yes	123 (53.01%)	88 (48.09%)	35 (71.43%)	
No	109 (46.98%)	95 (51.91%)	14 (28.57%)	
LNM rate (%)				<0.001^*^
Median	27.3	22.7	45.9	
Range	0–100	0–100	0–100	
N stage				<0.001^*^
N0	24 (10.34%)	23 (12.57%)	1 (2.04%)	
N1a	92 (39.66%)	84 (45.90%)	8 (16.33%)	
N1b	116 (50.00%)	76 (41.53%)	40 (81.63%)	
Cumulative RAI dose (mCi)				<0.001^*^
Median	100	30	150	
Range	30–800	30–270	30–800	

N stage was evaluated according to the eighth Tumor–Node–Metastasis Classification of the American Joint Committee on Cancer (16).

NED, no evidence of disease; PRD, persistent/recurrent disease; LNM, lymph node metastasis.

^*^P value <0.05.

### Predictors for persistent/recurrent disease

During follow-up, seven patients presented with continuous TgAb levels >4,000 IU/mL, of which four remained NED, one had persistent and progressive lung metastatic lesions, and two had cervical lymph node recurrences.

Among 225 patients whose ΔTgAb could be calculated, 179 presented as NED, while 46 exhibited as PRD. In the PRD group, 24 patients had a persistent disease (nine in the cervical lymph nodes, 12 in the lungs, and three in both) and 22 had a recurrent disease (16 in the cervical lymph nodes, four in the lungs, and two in both). Moreover, progressive diseases were observed in 27 cases (16 in cervical lymph nodes, nine in the lungs, and two in both). We performed binary logistic regression analyses to assess these variables’ potential impact on the presence or absence of persistent/recurrent DTC (**Table 2**). By univariate logistic analysis, several risk factors including larger tumor size, capsular invasion, high LNM rate, advanced N stage, high cumulative RAI dose, and low ΔTgAb were significantly associated with PRD. Considering that a higher cumulative RAI dose is commonly recommended for those with a high risk of either mortality or recurrence, patients requiring high therapeutic doses are more likely to have a persistent/recurrent disease, such as lung metastases. Thus, the cumulative RAI dose was only included in the multivariate regression as a corrective variable for confounders. According to the multivariate logistic analysis, LNM rate (*P* = 0.009, OR = 1.044, 95% CI: 1.011–1.079) and ΔTgAb (*P* < 0.001, OR = 0.948, 95% CI: 0.929–0.967) were independent risk factors of PRD.

**Table 2:** Univariate and multivariate logistic regression analyses for factors associated with persistent/recurrent disease.

Variables	Univariate analysis			Multivariate analysis		
	OR	95% CI	*P* value	OR	95% CI	*P* value
Age at diagnosis	1.004	0.973–1.037	0.797			
Gender	1.117	0.491–2.544	0.791			
Histology	0.972	0.106–8.913	0.980			
Maximal tumor size	1.407	1.021–1.939	0.037^*^	1.107	0.536–2.285	0.784
Bilaterality	1.032	0.522–2.038	0.928			
Multifocality	1.504	0.783–2.888	0.221			
Hashimoto’s thyroiditis	1.053	0.549–2.017	0.877			
Capsular invasion	2.528	1.264–5.056	0.009^*^	1.245	0.320–4.844	0.751
LNM rate	1.037	1.021–1.053	<0.001^*^	1.044	1.011–1.079	0.009^*^
N stage			<0.001^*^			0.791
N1a vs. N0	2.190	0.260–18.422	0.470			
N1b vs. N0	11.819	1.535–90.989	0.018^*^			
Cumulative RAI dose	1.016	1.010–1.021	<0.001^*^	1.022	1.011–1.033	<0.001^*^
ΔTgAb	0.951	0.937–0.965	0.001^*^	0.948	0.929–0.967	<0.001^*^

ΔTgAb, decrease percentage of TgAb level; OR, odds ratio; CI, confidence interval. *P value < 0.05.

### ROC analysis to predict persistent/recurrent disease

**Figure 2:**
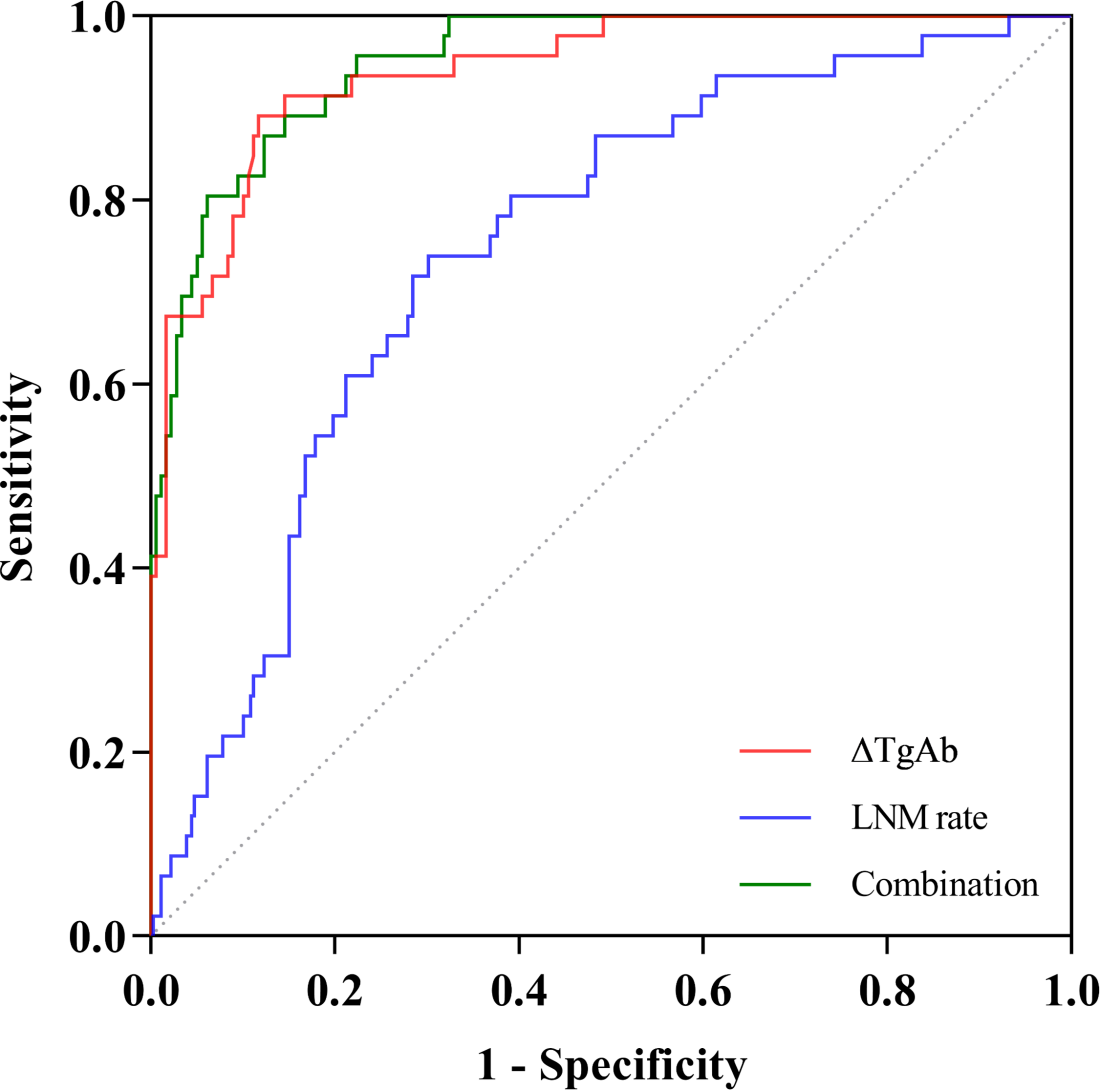
ROC curves of LNM rate, ΔTgAb, and their combination in predicting persistent/recurrent disease.

As shown in **Figure 2**, we plotted ROC curves incorporating ΔTgAb, LNM rate, and their combination. The optimal cutoffs of LNM rate and ΔTgAb in predicting PRD were 35.1% (*P* < 0.001, AUC = 0.745, 95% CI: 0.683–0.801) and 47.0% (*P* < 0.001, AUC = 0.940, 95% CI: 0.900–0.967), respectively. The AUC of the combination of both (LNM rate and ΔTgAb) was 0.950 (*P* < 0.001, 95% CI: 0.913–0.975). Their diagnostic performance indexes are displayed in **Table 3**, of which ΔTgAb >47.0% had the highest negative predictive value (NPV) of 96.93%. Furthermore, a comparison of the AUCs by DeLong’s test showed that ΔTgAb alone and the combination had a significantly greater predictive power than that of LNM rate (both *P* < 0.001), with no statistical difference between ΔTgAb alone and the combination observed (*P* = 0.203) (**Table 4**).

**Table 3:** ROC curve analysis and diagnostic performance.

Variables	AUC (95% CI)	Optimal cutoff	Sensitivity (%)	Specificity (%)	PPV (%)	NPV (%)
LNM rate	0.745 (0.683–0.801)	35.1%	71.74	71.51	39.20	90.79
ΔTgAb	0.940 (0.900–0.967)	47.0%	89.13	88.27	66.12	96.93
Combination	0.950 (0.913–0.975)	18.1%	86.96	87.71	64.45	96.33

AUC, area under the ROC curve; PPV, positive predictive value; NPV, negative predictive value.

**Table 4:** Pairwise comparisons among the AUCs of LNM rate, ΔTgAb, and the combination.

Comparisons of variables	Difference between areas (95% CI)	*Z* test	*P* value
ΔTgAb vs. LNM rate	0.194 (0.110–0.279)	4.496	<0.001^*^
LNM rate vs. combination	0.205 (0.130–0.280)	5.331	<0.001^*^
ΔTgAb vs. combination	0.011 (-0.006–0.027)	1.273	0.203

*P value < 0.05.

### Quantitative changes of TgAb levels in different disease status

**Figure 3:**
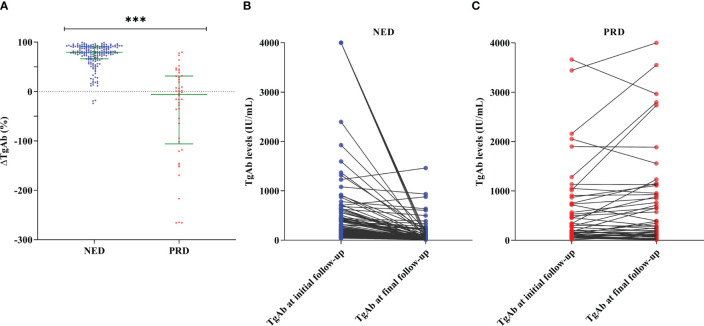
Changes of TgAb levels in patients with different disease status of differentiated thyroid cancer. **(A)** Comparison of ΔTgAb by Mann–Whitney test showed that ΔTgAb was higher in the no evidence of disease (NED) group than in the persistent/recurrent disease (PRD) group (*P* < 0.001). Trends of TgAb levels from initial to final follow-up of 179 patients with NED **(B)** and 46 patients with PRD **(C)**. Of the 28 patients with increasing TgAb levels, 25 (89.29%) were PRD and the other three remained NED. ***P value < 0.001.

Excluding seven patients whose ΔTgAb could not be calculated due to consistent TgAb levels above the upper limit of detection (>4,000 IU/mL), changes of TgAb levels during follow-up in patients with different disease status are illustrated in **Figure 3**. Among 179 patients with NED (**Figure 3B**), 176 (98.32%) demonstrated a spontaneous decrease in TgAb levels during follow-up, 158 (88.27%) had a decrease >47.0%, 130 (72.63%) had a decrease below 40 IU/mL, and only three (1.68%) showed progressively increasing TgAb levels. Among 46 patients with PRD (**Figure 3C**), 25 (54.35%) exhibited a gradually increasing trend of TgAb over time, 21 (45.65%) had decreasing TgAb levels, five of whom (10.87%) had TgAb levels below 40 IU/mL at the latest visit, without any significant increase in Tg.

### Effects of LNM rate and different TgAb changes on progression-free survival

**Figure 4:**
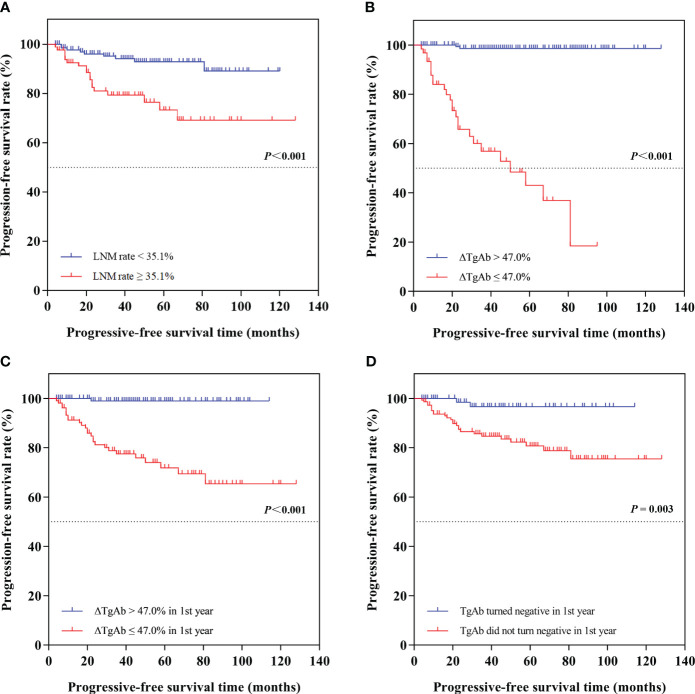
Kaplan–Meier curves of progression-free survival (PFS) in different groups of differentiated thyroid cancer patients. The log-rank analyses and Kaplan-Meier curves showed **(A)** patients in the LNM rate < 35.1% group had longer PFS than those in the LNM rate ≥ 35.1% group (P < 0.001); **(B)** patients in the ∆TgAb > 47.0% group had longer PFS than those in the ∆TgAb ≤ 47.0% group (not reached vs. 50 months, P < 0.001); **(C)** patients in ∆TgAb > 47.0% in the first year had longer PFS than those in the ∆TgAb ≤ 47.0% group (P < 0.001); **(D)** patients who turned negative in the first year had longer PFS than those who did not turn negative in the first year (P = 0.003).

According to the optimal cutoff value of LNM rate, we divided the patients into two groups of LNM rate ≥35.1% and LNM rate <35.1% for further analyses. The log-rank analysis and Kaplan–Meier curve showed a significant difference of PFS between the two groups (**Figure 4A**): patients in the LNM rate <35.1% group had longer PFS than those in the LNM rate ≥35.1% group (*P* < 0.001).

Based on the optimal cutoff value of ΔTgAb, we divided the patients into two groups: ΔTgAb ≤47.0% and ΔTgAb >47.0%. During follow-up, progressive disease was found in 40.32% (25/62) of patients with ΔTgAb ≤47.0%, and only 1.22% (2/163) had ΔTgAb >47.0%. Additionally, the log-rank analysis and Kaplan–Meier curve showed that patients in the ΔTgAb >47.0% group had longer PFS than those in the ΔTgAb ≤47.0% group (not reached vs. 50 months, *P* < 0.001) (**Figure 4B**). Considering the time-dependent nature of TgAb, we grouped the decrease rate and status of TgAb by 1-year cutoff. Of the 225 patients with calculable ΔTgAb, 117 patients had a decrease >47.0% of TgAb in the first year, with a median time of 7 months; the other 108 patients had a decrease ≤47.0% in the first year, and the former had a longer PFS than the latter (*P* < 0.001), as shown by the survival curve in **Figure 4C**. Furthermore, 135 patients achieved TgAb negative conversion (TgAb dropped to below 40 IU/mL) at final follow-up, with a median time of 8 months. Among these, 78 patients became negative during the first year, 57 became negative after the first year, and 90 patients remained positive at the end of follow-up. The survival curve showed that patients who turned negative in the first year had a longer PFS than those who did not turn negative in the first year (*P* = 0.003) (**Figure 4D**).

## Discussion

The management of DTCs with positive TgAb has been a challenge due to its measuring interference of Tg in up to one-fourth of the patients. The focus of this study was to explore the clinical value of TgAb change during follow-up to predict different disease status in those TgAb-positive DTC patients and estimate whether it might be used as an indicator for physicians to timely adjust the follow-up management. Different from the median age at diagnosis of 51 years among thyroid cancer patients revealed by Surveillance, Epidemiology, and End Results (SEER) statistics and the male-to-female incidence ratio of approximately 1:3, in our cohort with TgAb-positive, the patients were much younger, with a median diagnosis age of 34 years old, and have a much lower male-to-female incidence ratio as 1:4.66 (41 vs. 191) (17, 18). These discrepancies may suggest that female patients are more likely to suffer autoimmune thyroiditis (AT) and a tendency of early onset of thyroid cancer derived from this immuno-inflammatory background, which are quite similar as the findings shown by others (19, 20). A recent prospective cohort study that included 9,851 patients with thyroid nodules also noted an increased risk of malignancy associated with Hashimoto’s thyroiditis (HT), which suggested that thyroiditis might lead to tumor formation in a similar way that chronic inflammation of many tissues leads to cancer development (21). We thus assumed that young people and women may be more susceptible to underlying thyroid disorders (e.g., Hashimoto’s thyroiditis), which might accelerate the development and lead to their earlier onset of DTC by the change of thyroid function, particularly elevated TSH and inflammatory stimulation that adversely affects normal thyroid tissue. All these may remind us that thyroiditis and TgAb-positive might be a potential pre-cancerous sign which should be taken more seriously, especially in young and female patients. Studies have been performed to evaluate the tumor characteristics and prognosis of DTC patients with Hashimoto’s thyroiditis. A multicenter study including 301 intrathyroidal PTC assessed the relationship between HT and disease outcome. The results showed that HT was detected in 42.5% of patients and was associated to female gender, smaller tumor size, lower incidence of aggressive PTC variants, and less frequent radio-iodine administration. Besides these, HT was associated with a significantly higher clinical remission rate, and recurrence-free survival (RFS) was significantly longer in PTCs with HT compared to non-HT tumors (22). Another multicenter study prospectively collected 4,233 DTC patients, of whom 36.7% had autoimmune thyroiditis. By comparing the clinical characteristics and outcomes of patients with or without AT at 1-year follow-up, they observed that AT patients were significantly younger and had a smaller and bilateral tumor. In addition, AT patients had more biochemical persistence disease, but there was no significant association between the presence of AT and the structural persistence disease, the potential explanation being the ability of the residual thyroid tissue of AT patients to continuously secrete TgAb, thus leading to an incomplete biochemical response (23). These results indicated that the presence of HT might only cause serological abnormalities in TgAb during clinical follow-up, but not related to the persistence or occurrence of structural disease, and might even be associated with better clinico-pathological features and prognosis. In our study, a higher percentage of 54.31% in TgAb-positive DTC patients with HT was present, illustrating that some of TgAb are probably induced by HT. We used structural persistent/recurrent disease as primary endpoint, and the result of the univariate logistic regression analysis showed no significant association between Hashimoto’s thyroiditis and structural persistent/recurrent DTC in the follow-up (*P* = 0.877). Our study mainly predicted structural disease by serum TgAb changes, and since both suspected structural lesions and unsatisfactory biochemical levels provide evidence of disease persistence/recurrence, it is noteworthy that in the clinical follow-up of DTC we should not only monitor the occurrence of structural disease by imaging techniques but also focus on the serological changes at the same time for a more comprehensive assessment of persistent/recurrent disease.

In cases whose thyroid tissue and Tg antigen are removed by surgery and RAI ablation without persistent or recurrent disease, TgAb concentrations have always been found to decrease over months to years and eventually disappear (24). Therefore, the normal upper limit of 115 IU/mL obviously cannot be regarded as the negative cutoff of TgAb in those with thyroid removal by surgery and RAI ablation. Thus, in this study, a cutoff value of 40 IU/mL was taken, as it has been applied in several studies to qualitatively diagnose TgAb status (positive or negative) (14, 15). Numerous studies have demonstrated that the trend of TgAb after initial treatment might serve as a surrogate predictor of a disease-free status of no residual disease (especially when TgAb is remarkably decreasing) or the presence of structural disease (especially when TgAb is *de novo* or gradually increasing) (6–11, 13, 25, 26). Meanwhile, there is still controversy concerning the association between TgAb change and DTC disease status. Studies have shown that the trends of TgAb levels could not predict the disease status of DTCs, manifested as either a similar proportion of recurrent cases in the negative trend (slope < 0) group to that in the positive (slope > 0)/no trend (slope close to 0) group based on linear regressions of TgAb values from the surgery date until the end of follow-up or no statistically significant association between *de novo* TgAb development and structural recurrence (27, 28). So far, few studies have quantitatively analyzed the dynamic changes of TgAb in adult DTCs and mostly limited to analyze its association with the efficacy of RAI ablation. Thus, in this study, TgAb changes were used and quantitatively assessed to associate with disease status in terms of recurrence/persistence or not, while the well-accepted response to therapy assessment was not used since TgAb-positive DTCs are poorly defined by such. After adjusting for other possible clinical predictors (age at diagnosis, gender, histology, maximal tumor size, bilaterality, multifocality, Hashimoto’s thyroiditis, capsular invasion, N stage, and cumulative RAI dose), we obtained two independent variables including ΔTgAb and LNM rate, which could sensitively reflect DTC disease status during follow-up. ΔTgAb was identified to be negatively associated with persistent/recurrent DTC, while LNM rate was positively associated with such. Our finding suggests that ΔTgAb, an index derived from dynamic serial TgAb monitoring, could be an objective marker to sensitively reflect the disease status over time. As for LNM rate, although it reflects more about the thorough LN assessment at surgery, it also correlates with the degree of lymph node invasion. A high LNM rate denotes that the patient has a more aggressive tumor with a large number of lymph node involvement and may be relatively susceptible to the presence of PRD during follow-up, implying that more active management such as RAI therapy should be considered in such patients. From the ROC curve analysis, a high predictive value for DTC disease status was revealed among all of the independent risk factors including ΔTgAb, LNM rate, and their combination. Though the AUC for the combination was the highest and significantly greater than that for LNM rate, there was no significant difference between the combination and ΔTgAb alone. Therefore, ΔTgAb alone could be applied as a convenient and user-friendly marker during follow-up, holding a high diagnostic value with sensitivity and specificity of 89.13% and 88.27%, respectively. It is impressive that the NPV of ΔTgAb in our study was up to 96.93%, suggesting that ΔTgAb could be used as an excellent negative indicator for disease-free status to avoid over-management in those with ΔTgAb >47.0%, which appeared more objectively than the cutoff of 50% raised arbitrarily by experience in several studies (7, 9, 10, 29, 30).

According to a retrospective study of 824 DTC patients by Kim et al. (7), serum TgAb levels measured at 6–12 months after remnant ablation could predict recurrence in TgAb-positive patients. We compared different TgAb decrease rates and status in PFS by 1 year as a boundary and found that patients with ΔTgAb >47.0% in the first year had a longer PFS than those with ΔTgAb ≤47.0%. Another result that we obtained from the survival curve was that patients who turned TgAb-negative in the first year had a longer PFS than those who did not turn negative. These results implied that the decrease rate and status of TgAb in the first year after RAI treatment could serve as early indicators for predicting disease-free survival in TgAb-positive patients. Of the 135 patients who dropped to below 40 IU/mL at final follow-up, the median time to achieve negative conversion was 8 months, which was similar to our prior study (31). For those with positive TgAb and failed to turn negative, one potential explanation might be the relatively higher level of TgAb prior to ^131^I therapy, which may take a longer time to achieve negative conversion; thus, a longer follow-up for such patients in this study is still ongoing. For patients with rapidly rising or even consistently positive but stable TgAb, one recently published consensus warns of the risk that such patients may carry a high suspicion of PRD (32). A meta-analysis including 34 studies also reported that patients with persistent/increasing TgAb levels had a higher risk of PRD (12), which was also the case in our study. Nearly 90% (89.29%, 25/28) of the 28 patients with increasing TgAb levels were proved to be PRD. Thus, in patients with rising TgAb, more active surveillance, including both serological and imaging diagnostic procedures, is required. It is worth noting that from the Kaplan–Meier curves, we can see as long as 50 months of PFS even in patients with a slow decrease of TgAb, reminding that a more patient and longer follow-up is needed for such patients. It is noteworthy that, in the present study, the value of ΔTgAb in identifying persistent/recurrent disease of DTC seems less promising, with a PPV less than 70% (66.12%). This may be due to the indolent nature of DTC and the relatively short follow-up time of our study, which might have limited its power to identify PRD. Additionally, 21 of 46 (45.65%) PRD patients had decreasing TgAb levels, and five (10.87%) turned out TgAb-negative before the latest visit. One potential explanation is the time delay of TgAb change, with PRD occurring within a relatively short follow-up time after treatment while TgAb is still in a period of decrease. Alternatively, some PRD patients may present an iodine refractory status of tumor dedifferentiation, which reduces the capacity of the lesion to secrete Tg, resulting in TgAb maintained at a low level (33). Under this circumstance, additional imaging such as US, CT, or ^131^I WBS and PET/CT may be more complementary in identifying those with persistence or recurrence. Besides these, in this study, patients with TgAb levels consistently above the upper limit of detection were noticed not only in the PRD group (3/7) but also in the NED group (4/7), which has also been reported by Chiovato (6). We propose that it may be due to several scenarios as follows: there may still be undetected tumor foci or related to immuno-thyroiditis background or the existence of long-lived memory cells that continue to produce TgAb in the patient’s body (3). In such cases, a longer follow-up and a high-sensitivity examination for the detection of potential foci are necessary.

Several limitations in our study should be mentioned. First, the number of PRD cases was limited, which may result in a low PPV, and a larger sample size is required to confirm our findings in future studies. Second, due to ethical concerns, some of the PRD cases, particularly distant metastases, were diagnosed merely based on typical imaging features and anatomical changes in their follow-up, without having been pathologically confirmed. Additionally, the follow-up period of our study was relatively short, a more long-term follow-up is required to verify the predictive value of ΔTgAb over time.

## Conclusion

In summary, our study suggested that ΔTgAb could serve as a valuable indicator of disease status in DTC patients with positive postoperative TgAb. ΔTgAb of >47.0% is conducive to identify those with NED to obviate their overtreatment. The decrease rate and negative conversion of TgAb in the first year were good predictors of disease-free survival in patients.

## Data availability statement

The original contributions presented in the study are included in the article/**Supplementary Material**. Further inquiries can be directed to the corresponding author.

## Ethics statement

The studies involving humans were approved by Ethical Committee of Peking Union Medical College (PUMC) Hospital. The studies were conducted in accordance with the local legislation and institutional requirements. The human samples used in this study were acquired from primarily isolated as part of your previous study for which ethical approval was obtained. Written informed consent for participation was not required from the participants or the participants’ legal guardians/next of kin in accordance with the national legislation and institutional requirements.

## Author contributions

YZ: Writing – review & editing, Writing – original draft, Visualization, Validation, Supervision, Project administration, Methodology, Investigation, Formal analysis, Data curation, Conceptualization. ZM: Investigation, Data curation, Writing – review & editing, Visualization, Validation, Supervision, Methodology, Formal analysis, Conceptualization. DL: Investigation, Writing – review & editing, Visualization, Validation, Methodology, Formal analysis, Data curation. TZ: Writing – review & editing, Conceptualization, Visualization, Validation, Methodology, Formal analysis, Data curation. XZ: Writing – review & editing, Supervision, Validation, Investigation, Data curation, Conceptualization. DS: Writing – review & editing, Validation, Supervision, Formal analysis, Methodology, Investigation. YS: Writing – review & editing, Supervision, Conceptualization, Methodology, Data curation. JL: Writing – review & editing, Visualization, Methodology, Validation, Supervision. YL: Methodology, Writing – review & editing, Validation, Supervision, Conceptualization.
